# Use of a steerable microcatheter during superselective angiography: impact on radiation exposure and procedural efficiency

**DOI:** 10.1186/s42155-019-0078-9

**Published:** 2019-11-04

**Authors:** Jason C. Hoffmann, Jonathan Minkin, Nicholas Primiano, Jung Yun, Abieyuwa Eweka

**Affiliations:** 1 Department of Radiology, NYU Winthrop Hospital, 259 First Street, Mineola, New York, 11501 USA; 2 Stony Brook School of Medicine, 101 Nicolls Road, Stony Brook, New York, 11794 USA; 3 State University of New York at Buffalo Jacobs School of Medicine and Biomedical Sciences, 955 Main Street, Buffalo, New York, 14203 USA

**Keywords:** Selective angiography, Steerable microcatheter, Radiation exposure, Procedure efficiency

## Abstract

**Background/purpose:**

To study steerable microcatheter (SM) use in moderate and highly difficult vessel selection compared to conventional pre-shaped microcatheter (CM) use.

**Material and methods:**

An IRB approved, single institution analysis of 40 complex angiographic procedures with and without superselective microcatheter use during an eight-month period in 2017 was performed. Target vessels were deemed moderate or highly difficult to select based on vessel size, tortuosity, and/or angulation during non-selective initial angiography. Data collected included type of microcatheter used (SM or CM), number of microcatheters and microwires used, procedure time, radiation exposure index (dose area product/DAP), target vessel location, and time to target vessel selection (TTVS; time from device placement to vessel selection). Comparison between the SM and CM groups was performed using Wilcoxon test.

**Results:**

A SM (SwiftNinja, Merit Medical, South Jordan, UT, USA) was used to select 46 vessels in 20 patients. One or more CMs were used in 20 patients to select 34 vessels. Median TTVS, number of microwires used, total procedure time, and DAP (microGray^.^m^2^) were 12 vs. 462.5 s (*p* < 0.0001), 0 vs. 2 (*p* < 0.001), and 26,948 vs. 30,904 (*p* = 0.15) in the SM vs. CM groups, respectively. When adjusted for body mass index (BMI) using a linear model for radiation exposure, patients in the SM group had lower radiation exposure than those in the CM group (*p* = 0.05).

**Conclusions:**

Utilization of a steerable microcatheter, without or with a guidewire, leads to easier and faster target vessel selection with shorter procedure times in complex vessel anatomy.

## Introduction

Until recently, all superselective angiography and transartertial embolization procedures were performed with a variety of conventional microcatheters and guidewires. Conventional microcatheters have a pre-determined shape that cannot be changed while inside of a patient. However, a new option has recently been introduced. The SwiftNINJA® steerable microcatheter (Merit Medical, South Jordan, UT, USA) received FDA 510(k) clearance in November 2016, and is the first and only steerable microcatheter currently available in the United States (https://www.accessdata.fda.gov/scripts/cdrh/cfdocs/cfpmn/pmn.cfm?ID=K161921 2018). This microcatheter allows users to change the angle and shape of its tip in real-time while the device is inside of a patient via a dial on the handgrip of the device (http://cloud.merit.com/catalog/IFUs/401719002_001.pdf 2018). It is designed to be used either with or without a guidewire (http://cloud.merit.com/catalog/IFUs/401719002_001.pdf 2018). The steerable microcatheter has a straight tip in the neutral position and can articulate up to 180 degrees in opposing directions. It has a 2.4F (0.80 mm) outer diameter at the tip, an inner diameter of 0.54 mm, and is 125 cm in length. The maximum particle size that can be delivered through the steerable microcatheter is 700 *u*m and the maximum coil size is 0.018-in. (http://cloud.merit.com/catalog/IFUs/401719002_001.pdf 2018).

Larger steerable catheters have been available for years and have been used in cardiovascular angiography. Prospective studies comparing these larger steerable catheters to standard catheters have showed decreases in failure rate, procedure time, fluoroscopy time, and amount of contrast required (Er et al. 2015; Wang et al. 2014). However, there are no comparable studies of the benefits of steerable microcatheters. A literature search revealed two papers describing three case reports (Soyama et al. 2017; Hinrichs et al. 2017), one animal study, and one clinical trial. In Inaba et al. 2017, the steerable microcatheter was compared to conventional microcatheters in animal models. They found decreased procedure times, fluoroscopy times, and contrast usage when using a steerable microcatheter (Inaba et al. 2017). Another study by Inaba et al. evaluated steerable microcatheter use in humans. They successfully cannulated 99% of the targeted vessels and did not require a guidewire to cannulate 98% of the vessels. No comparison was made to conventional microcatheters and no serious adverse events were noted (Inaba et al. 2018).

The real-time ability to change the degree of angulation of the tip of the steerable microcatheter has potential to improve efficiency of superselective vessel cannulation. The purpose of this study is to compare the use of steerable microcatheters (SM) to pre-shaped conventional microcatheters (CM) in moderate to highly difficult vessel selection by measuring time to target vessel selection (TTVS), procedure time, radiation exposure index, and ease of vessel selection.

## Materials and methods

After receiving Institutional Review Board approval, a single institution prospective analysis was performed from April through August 2017 of 18 cases selected for use of the steerable microcatheter (SM). This device was used due to moderate-to-high expected difficulty of cannulation based on vessel location, size, tortuosity, and/or angulation during periprocedural non-selective angiography via a 5-French base catheter. Additionally, two cases were selected for use of the steerable microcatheter due to failure of vessel selection with conventional microcatheters. These were retrospectively compared to 20 cases performed from January through April 2017 performed with conventional microcatheters (CM) with similar target vessel locations and expected difficulty of cannulation. CM used include Renegade (Boston Scientific Corporation, Marlborough, MA, USA) and Maestro (Merit Medical, South Jordan, UT, USA). Microwires utilized included 0.014″ shapeable guidewire (Fathom, Boston Scientific Corporation, Marlborough, MA, USA) and 0.018″ pre-shaped guidewires (90-degree and double-angle Glidewire GT, Terumo Interventional Systems, Somerset, NJ, USA). All procedures were performed at one institution by a single interventional radiologist with 7 years of attending-level experience. Before the beginning of this study, the interventional radiologist had performed three cases using the steerable microcatheter.

**Fig. 1:**
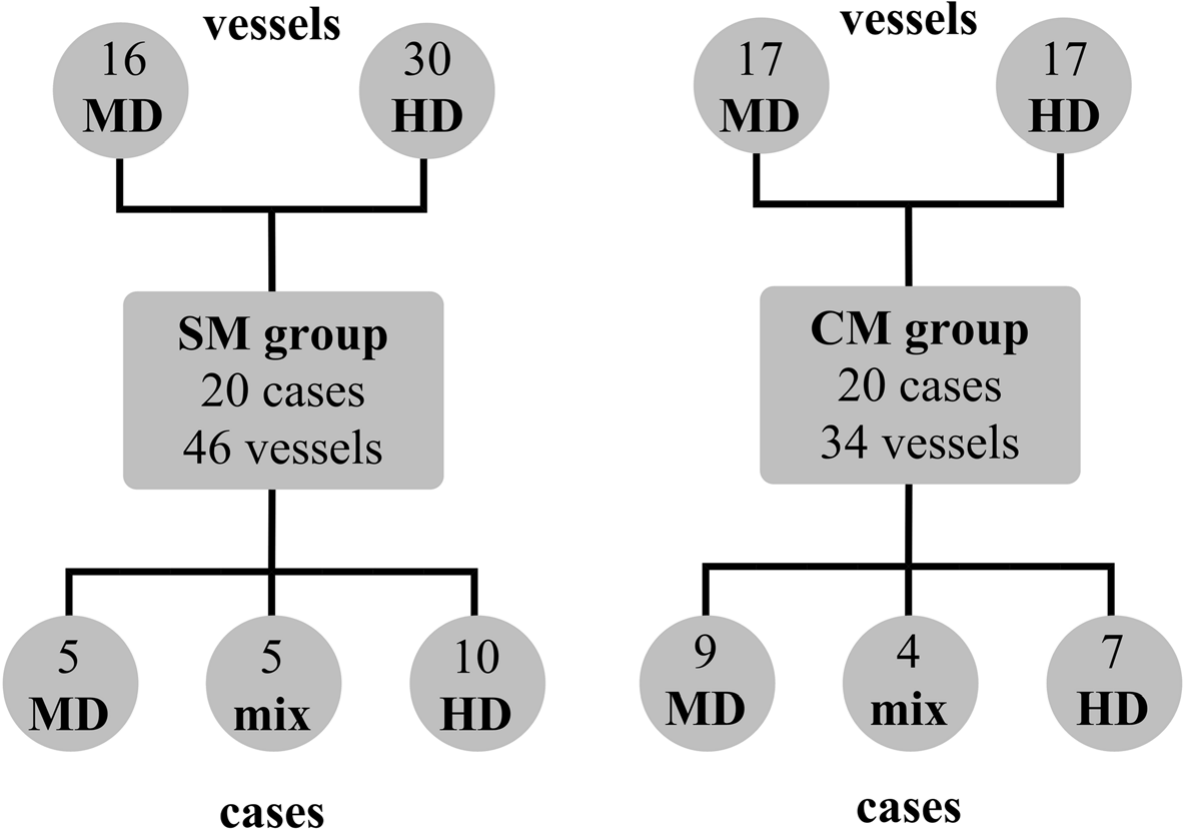
Breakdown of SM and CM cases into MD/HD/mix subgroups and all SM and CM vessels into MD or HD subgroups

The vessels targeted were classified into one of two subgroups based on location. Examples of moderate difficulty (MD) subgroup vessels include left, right, proper, and middle hepatic arteries, bronchial arteries, and gastroduodenal arteries. High difficulty (HD) subgroup vessels include a vessel with multiple 90-degree (or greater) curves, acute angle of origin off of feeding vessel, and/or origin arising at area of 90-degree or greater curve of the feeding vessel. Examples include third-order branches of hepatic arteries, distal branches of the superior mesenteric artery (SMA), distal branches of renal arteries, vesicular arteries, prostatic arteries, distal branches of internal iliac arteries, and gastric arteries. In addition, two other cases were classified as HD: a uterine artery embolization in which selection had failed with conventional microcatheters and the cannulation of a particularly tortuous gonadal artery. Cases in the SM and CM groups were also subdivided into those involving only MD vessels, only HD vessels, or cases in which both types were cannulated (labeled as “mix” in Fig. 1). A summary of target vessels in the steerable microcatheter (SM) and conventional microcatheter (CM) groups are included in Table 1.

**Table 1:** Vessels cannulated in the SM and CM groups and their classification as either moderate-difficulty (MD) or high-difficulty (HD)

Target vessel	Subgroup	SM (n)	CM (n)
R/L/M/P hepatic	MD	15	13
Gastroduodenal	MD	1	3
Bronchial	MD	0	1
Branches of R/L hepatic	HD	7	3
Vesicular	HD	2	4
Renal artery branches	HD	3	0
SMA branches	HD	4	3
R/L gastric	HD	3	4
Uterine	HD	2	0
Gonadal	HD	1	0
Prostatic	HD	8	0
Internal iliac branches	HD	0	3

All procedures were performed via right common femoral artery access with mild to moderate intravenous sedation. In each case, the total time that elapsed during advancement of the microcatheter from the base catheter to the target vessel was measured using a timer on the angiography machine. This was recoded as “time to target vessel selection” (TTVS). In the prospective portion of this study (SM group), this was recorded during each case. In the retrospective portion of the study (CM group), this was calculated by using the time stamps on angiography and spot fluoroscopic stored images and technologist and nursing staff notes obtained during the case. To compensate for the initial time that it took to prepare the selected microcatheter and wire in the CM group, 120 s was subtracted from the time based on the information from time-stamps and other staff documentation (i.e., the time used for the study was 120 s less that the time noted from procedural documentation). Procedure start and end times were recorded by nursing staff and used to calculate total procedure time. The operator subjectively rated the expected difficulty of vessel cannulation based on preoperative angiography along with the actual difficulty experienced during the procedure using a 10-point Likert scale. Other information collected included the radiation exposure index (DAP: dose area product), contrast volume used, and the number of microcatheters and microwires used.

Statistical analysis was performed using the JMP statistical software package from SAS. A one-sided Wilcoxon rank-sum test was used to compare the TTVS of each vessel cannulated in the steerable microcatheter and conventional microcatheter cases. The same test was used to compare the procedure time, DAP, and contrast volume for each case. Then, the same test was used to separately compare HD cases and vessels in the steerable microcatheter and conventional microcatheter groups and to compare MD cases and vessels in the same groups.

## Results

Steerable microcatheters (SM) were used in 20 cases to select a total of 46 vessels. One or more conventional microcatheters (CM) were used in 20 cases to select a total of 34 vessels. Vessel cannulation was successful in all cases and there were no intraoperative or postoperative adverse events. No vessel spasm, dissection, or other vessel-related complications occurred in either group. In the SM group, no guidewires were used in 11 out of 20 cases and in selection of 33 out of 46 vessels. Cases using conventional microcatheters required at least one guidewire.

The time to vessel selection (TTVS) of the 46 vessels in the SM group (median 12 s, interquartile range 7–26 s) was found to be significantly lower than the 34 vessels selected in the CM group (median 462.5 s, interquartile range 50.5–921.25 s, *p* < 0.0001). Total procedure time was found to be significantly lower in the 20 cases of the SM group when compared to the 20 cases of the CM group (median 75 min compared to 112.5 min, *p* = 0.0107). Radiation exposure index measured as DAP was initially not found to be significantly different between the cases of the SM group and the cases of the CM group (median 26,948 microGray^.^m^2^ compared to 30,904.7 microGray^.^m^2^, *p* = 0.15). However, studies have shown that BMI is positively correlated with DAP (Galbraith et al. 2018; Ector et al. 2007). When adjusted for body mass index (BMI) using a linear model for radiation exposure, patients in the SM group had lower radiation exposure than those in the CM group (*p* = 0.05). The amount of contrast agent used was not found to be significantly different between the cases of the SM group and those of the CM group (median 87.5 mL compared to 80 mL, *p* = 0.50). Despite the SM group being perceived to have more difficult vessels to cannulate (median Likert score of 7 in SM group vs. 5 in CM group, *p* < 0.001), the actual ease of vessel cannulation perceived by the operator was lower/easier in the SM group (median Likert score of 3 in SM group vs. 5.5 in CM group, *p* < 0.001).

**Fig. 2:**
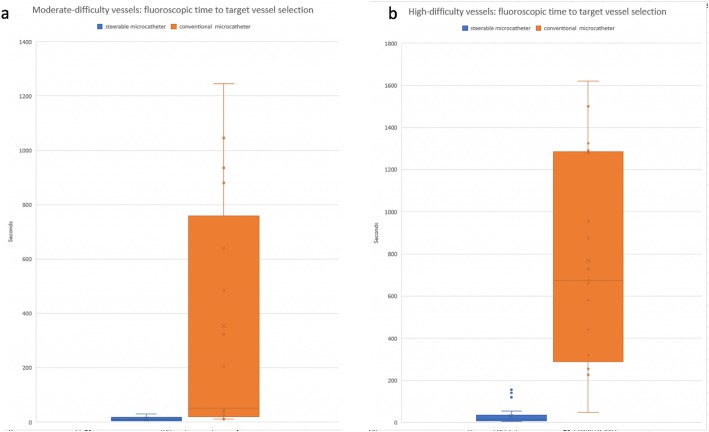
Distribution of time to target vessel selection in comparison of moderate difficulty (**a**) and high difficulty (**b**) cannulations with steerable microcatheter and conventional microcatheter use

**Fig. 3:**
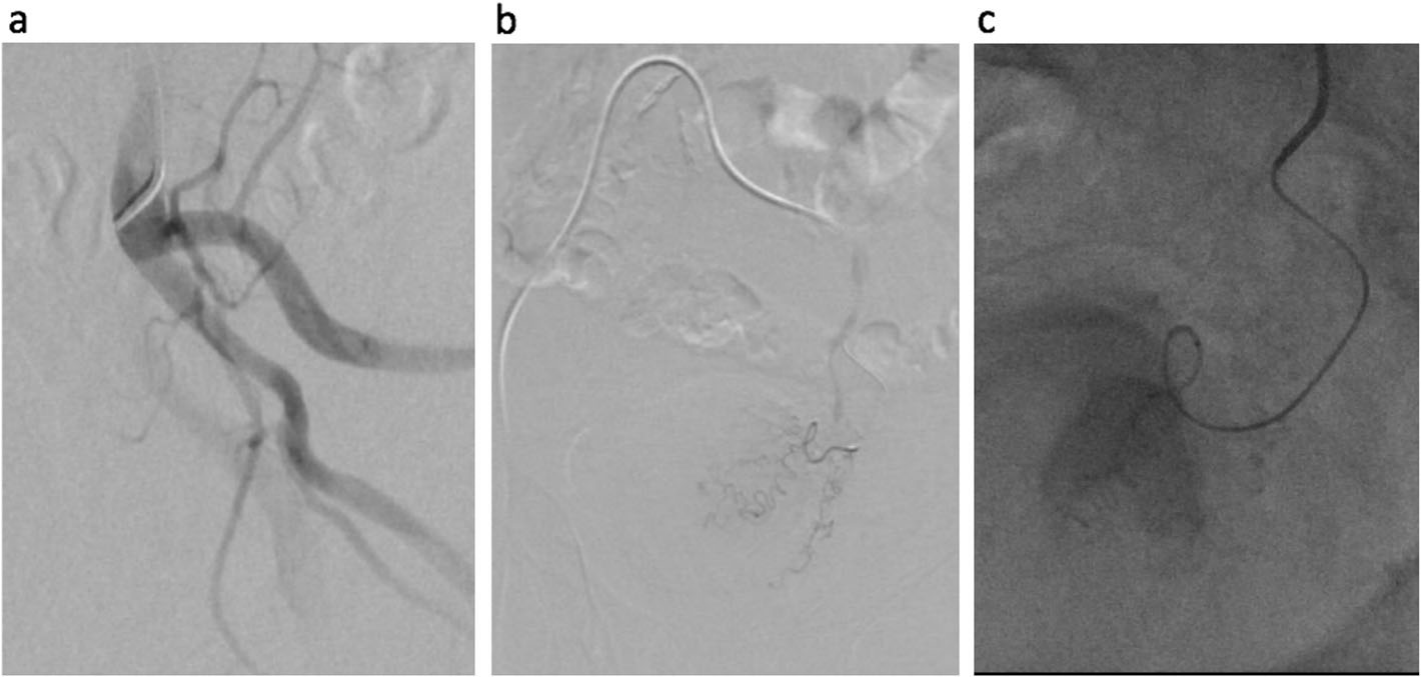
85 year-old male with Benign Prostatic Hyperplasia, severe lower urinary tract symptoms (IPSS 29), and 110 g prostate, who comes to interventional radiology for prostatic artery embolization. Left internal iliac angiography demonstrates common trunk of the superior and inferior vesical arteries (**a**). The left prostatic artery (PA) was selected with the SwiftNINJA® steerable microcatheter, with time to target vessel selection (microcatheter moved from tip of 5F catheter to successful left PA cannulation) of 12 s. No guidewire was needed. Left PA DSA demonstrates tortuous PA branches with no extra-prostatic enhancement (**b**). After initial relatively proximal left PA embolization, the steerability of the microcatheter was used to navigate through a tortuous 360-degree loop to gain more distal access (**c**), without the need for a guidewire. Additional distal embolization was then performed, following the “PErFecTED” technique. Patient was discharged home the same day. IPSS decreased to 8 within 1 month post procedure. Total procedure time for bilateral PAE was 100 min

**Fig. 4:**
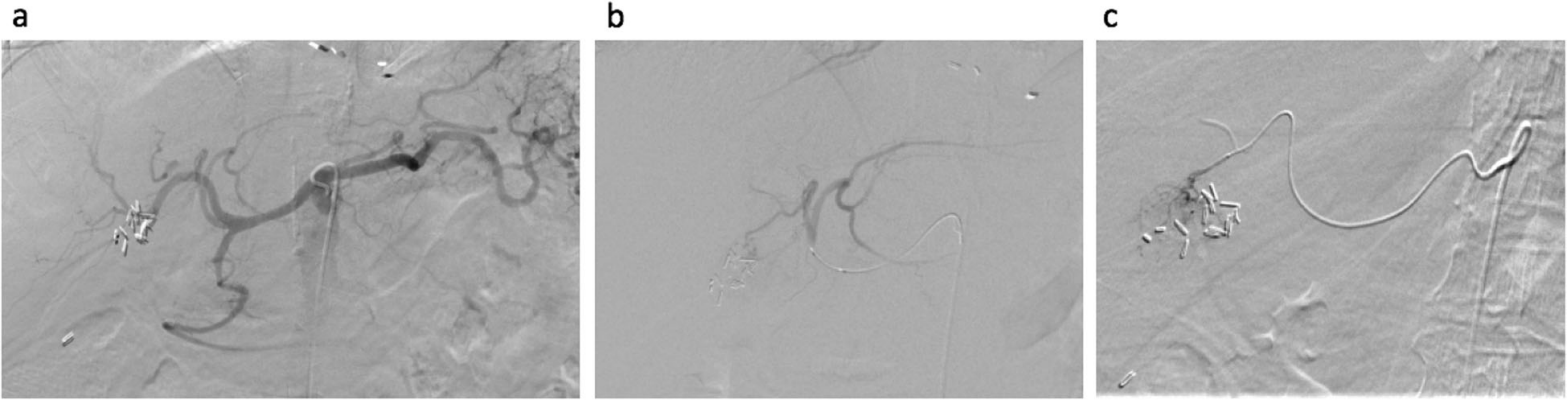
82 year-old female with segment 4a hepatocellular carcinoma. Recent myocardial infarction, treated with angiography and stent placement, thus must remain on aspirin and plavix. Celiac DSA demonstrates trifurcation of common hepatic artery into right hepatic, left hepatic, and gastroduodenal arteries (**a**). The SwiftNINJA® steerable microcatheter was used to select the left hepatic artery (LHA), and DSA images demonstrate a segment 4 branch artery off of the LGA supplying the tumor, with early arterial enhancement (**b**). The LHA was selected with the SwiftNINJA® and no guidewire, with time to target vessel selection of 9 s. The steerable microcatheter was used to superselect the branch artery supplying the HCC with prominent tumor enhancement (**c**). The time from SwiftNINJA® selection from proximal LHA to superselective branch vessel cannulation was 13 s, with no guidewire needed. After superselective transarterial chemoembolization, there was appropriate stasis in the feeding vessel. Total procedure time was 45 min

When separately comparing the MD vessels, the SM group was found to have lower TTVS (9 s compared to 52 s, *p* < 0.001). However, no significant differences were found in procedure time (median 60 min compared to 80 min, *p* = 0.25), DAP (median 18,655 microGray^.^m^2^ compared to 23,911 microGray^.^m^2^, *p* = 0.399), or contrast volume (55 mL compared to 75 mL, *p* = 0.21). When separately comparing the HD vessels, the SM group was found to have a lower TTVS (median 16.5 s compared to 675 s, *p* < 0.0001), lower procedure times (median 95 min compared to 145 min, *p* = 0.012), decreased DAP (25,815 microGray^.^m^2^ compared to 76,342 microGray^.^m^2^, *p* = 0.035), and decreased contrast volume (median 105 mL compared to 130 mL, *p* = 0.0228). Figure 2a and b which depict the distribution of TTVS in these subgroups. DAP was not corrected for BMI in the subgroup analysis. Case examples in the HD SM subgroup are included in Figs. 3 and 4.

## Discussion

In our study, the SwiftNINJA® steerable microcatheter was used for superselective angiography and embolization procedures. Target vessels were selected successfully in all cases and there were no adverse events reported. The device was found to be intuitive to use and a steep learning curve was not encountered. No guidewires were needed to cannulate most vessels. Even when selecting small and tortuous vessels, adequate steerability was achieved and the device tracked appropriately when used with a guidewire.

We found statistically significant improvements in time to target vessel selection which were most pronounced when cannulating high difficulty (HD) vessels. Vessels such as prostatic arteries and superior vesicular arteries are typically difficult and time consuming to cannulate and benefit the most from use of a SM that can better navigate tortuous vessels with variable anatomy. While no adverse events were noted in either group, fewer microcatheter exchanges and decreased time to vessel would be expected to be associated with a decrease in intraoperative complications such as vascular spasms and arterial dissection. Median total procedure time was found to be 37.5 min lower in the SM group, and 55 min lower in the high-difficulty subgroup. The time spent physically performing fluoroscopy and selecting the target vessel is one component of this. Further, less time with microcatheter and wire exchanges, angiography in different obliquities, and reviewing prior angiography and/or cross-sectional imaging studies to assess best approaches to vasculature may also have led to a significant decrease in overall procedure times. The increased throughput allowed by shorter procedure times and cost savings from the use of fewer microcatheters and guidewires may offset the higher cost of steerable microcatheters. Analysis of the cost-effectiveness of steerable microcatheters is a topic for future research.

After correcting DAP for BMI using a logarithmic transformation, SM use was associated with significantly lower DAP. In addition, when examining the high-difficulty subgroup, even uncorrected DAP was found to be significantly lower in the SM group. This suggests that decreased TTVS and procedure times is accompanied by a radiation safety benefit. A larger study would be beneficial for further evaluation. The volume of contrast used was also found to be lower with SM use, although only in the high difficulty subgroup. This can potentially impact nephrotoxicity, particularly in patients with borderline renal function.

**Fig. 5:**
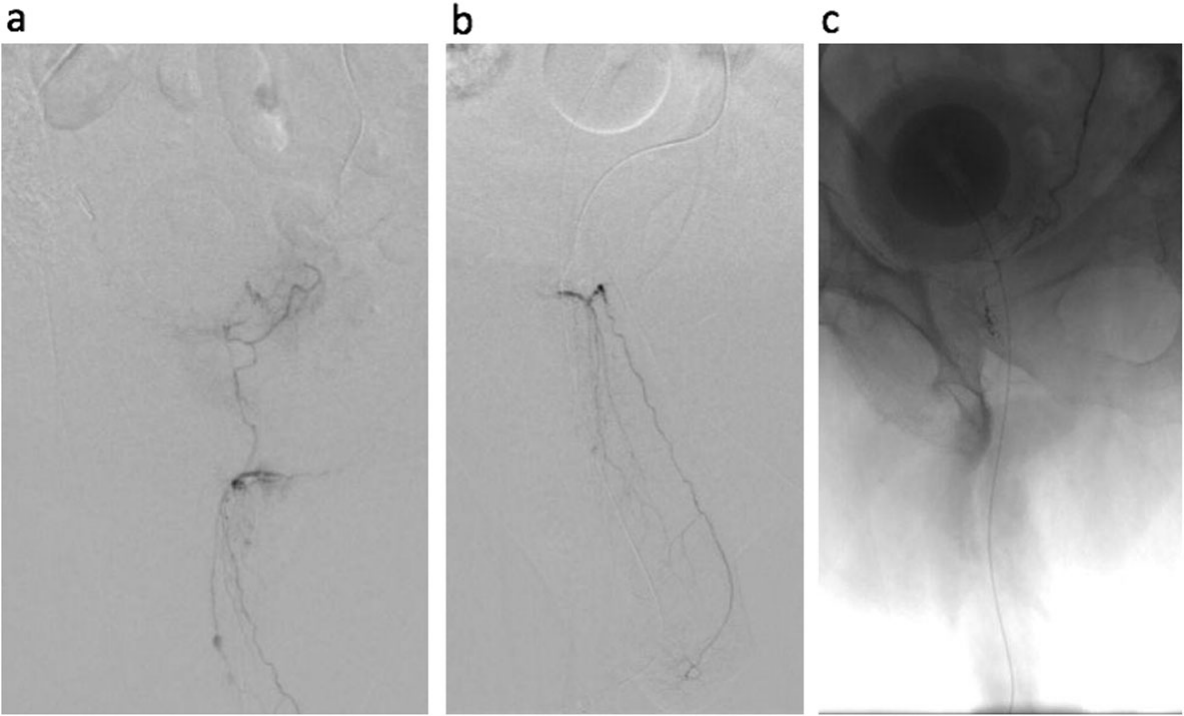
75 year-old male with BPH, prostate 75 g with IPSS 25 and QOL score of 5 at initial consultation, who developed urinary retention soon after initial PAE consultation. Steerable microcatheter used to select the left PA, and intra-prostatic collateral supply accessory blood flow to penis is present (**a**). The steerable microcatheter was used to select this collateral (**b**). Distal access was achieved, and a 2 mm diameter detachable microcoil was placed (**c**). The patient had successful PAE, with removal of the indwelling foley catheter 10 days after the procedure

The SM was used successfully in four prostatic artery embolization (PAE) procedures in patients with benign prostatic hyperplasia (BPH) experiencing lower urinary tract symptoms (LUTS). The PErFecTED technique was utilized (proximal embolization first, then followed by distal/central embolization), and bilateral embolization was successful in all four cases (Carnevale et al. 2014). The median time to advance the microcatheter from the internal iliac artery to the prostatic artery was 10.5 s. This is notable as PAE is considered to be a challenging procedure as prostatic artery anatomy is often variable, tortuous, and angulated. Further, as intra-prostatic collaterals require coil embolization in up to 20% of PAE cases, the ability to select and embolize these collaterals to prevent non-target embolization is important (Bhatia et al. 2018; Moreira 2017). The SM was able to be advanced into distal prostatic arterial vasculature bilaterally in each case, despite its 2.4F size. In addition, when needed, the SM can be placed into distal branch vasculature to allow for appropriate coil embolization (Fig. 5).

While the SM can be used as a bailout option during cases with more complex anatomy after extensive attempts with various pre-shaped microcatheters and microwires have failed, its use prior to opening multiple other devices has advantages in procedural efficiency, radiation exposure, and overall cost. The SM has a higher unit cost compared to fixed-shaped microcatheters; however, overall cost of use is relative. If the higher up front cost of the steerable microcatheter avoids the use of several lower cost microcatheters and microwires during a procedure, the per-case expenditure may be reduced. In addition, decreasing procedure times can allow for increased procedural efficiency and increased throughput in the angiography suite, with higher overall procedural volumes in a given period of time. The cost per minute to run and staff a procedure room is not insignificant, but does vary between institutions. Additional studies may be beneficial to further study this cost relationship.

Our study has multiple limitations. Case selection was non-randomized. In addition, the SM group was evaluated prospectively, while the CM group was evaluated retrospectively. When calculating TTVS in the control group, 120 s was subtracted from the time available based on time-stamps and other staff documentation to allow for initial time to prep the microcatheter and wire. This was chosen based on calculating the time it takes for the operator and staff to select and prep the equipment based on our experience, and over-estimated the prep time with the goal of minimizing risk of artificially higher TTVS in CM group based on prep time. Selection bias and interpretation bias can exist, given that a single operator performed all procedures. However, the cases assigned to the SM group appear to be more complex than those assigned to the CM group. In three cases, steerable microcatheters were only used after vessel cannulation had failed with conventional microcatheters. Cases in the SM group more often targeted two vessels while those in the CM group more often targeted just one. Also, there were more high-difficulty (HD) vessels in the SM group, including four bilateral PAE. However, additional studies at multiple institutions and with multiple operators would be beneficial for further investigation. In addition, no radial access cases were included as the steerable microcatheter currently is 125-cm in length, limiting its use from this access.

## Conclusion

Steerable microcatheter use was associated with improvements in time to target vessel selection and procedure time as compared to the conventional microcatheter. These improvements were most pronounced when cannulating vessels that are typically most time consuming and difficult to cannulate. Our findings suggest that steerable microcatheter use has a benefit in superselective angiography with positive impacts on procedural efficiency and radiation exposure. This can have relevance from both a radiation safety perspective (for patients, physicians, and IR staff) as well as a workflow perspective.

## Data Availability

The datasets analyzed during the current study are available from the corresponding author on reasonable request.

## Abbreviations

BMI: Body mass index
BPH: Benign prostatic hyperplasia
CM: Conventional microcatheter
DAP: Dose area product
HD: Highly difficult
LUTS: Lower urinary tract symptoms
MD: Moderately difficult
PAE: Prostatic artery embolization
SM: Steerable microcatheter
SMA: Superior mesenteric artery
TTVS: Time to target vessel selection
